# Prevalence of HBV genotypes among patients attending Moi Teaching and Referral Hospital liver clinic

**DOI:** 10.1371/journal.pone.0305753

**Published:** 2024-07-10

**Authors:** Caroline Wangui Gikunyu, Elius Mbogori, Arthur Kwena, Geoffrey K. Maiyoh

**Affiliations:** 1 Department of Biochemistry and Clinical Chemistry, School of Medicine, Moi University, Eldoret, Kenya; 2 Department of Laboratory Services, Moi Teaching and Referral Hospital, Eldoret, Kenya; 3 Department of Pathology, School of Medicine, Moi University, Eldoret, Kenya; Centre de Recherche en Cancerologie de Lyon, FRANCE

## Abstract

Hepatitis B virus (HBV) belongs to the genus Orthohepadnavirus, of Hepadnaviridae family, smallest human deoxyribonucleic acid (DNA) virus with 3200 bp in a partially double-stranded circular DNA. Globally, about 2 billion people are infected with over 65 million of the chronically infected residing in Africa. Ten HBV genotypes (A-J) have been reported across the globe. Based on the World Health Organization (WHO) African Regions including Kenya have high HBV prevalence rates yet the data on prevalence rates of the various HBV genotypes and their associated biomarkers is very scanty. A cross-sectional descriptive study with purposive sampling was conducted in which a census of patients with chronic Hepatitis B (CHB) with history >6-month were reviewed for eligibility. Demographics data was abstracted from patient files and blood samples drawn for genotyping, viral load using Rotor gene Q Polymerase Chain Reaction (PCR) equipment, Hepatitis B surface Antigen (HBsAg), Hepatitis B envelope antigen (HbeAg) and Hepatitis B core antibody (Anti-HBc) using Cobas e411 machine. Out of a total of 83 patients, 43 (52%) were eligible; males 29 (67.4%), females 14 (32.6%) with mean ages of 35.1±10.8 and 34.3±9.3 respectively. Genotypes A were 34(79.1%), B were 5(11.6%), C-D were 0 while E-J were 9(20.9%). All cases of genotype B were associated with co-infection of genotype A. Majority were HBeAg negative with HBV DNA >10 IU/ml (81.4% and 86.0% respectively) with distribution among all the genotypes. Across genotypes, viral load mean percentage comparisons were: A vs. A/B = 2600 (p = 0.09), A vs. E-J = 5260 (p = 0.09) and A/B vs. E-J = 200 (p = 0.28). The most prevalent genotype was A followed by mixed co-infection of genotype A/B. Genotype A was associated with HBV DNA viral loads > 10IU/ml and high rates of HBeAg negativity. Genotypes E-J were also detected though not characterized.

## 1 Introduction

Hepatitis B Virus belongs to the genus Orthohepadnavirus, of Hepadnaviridae family, which is the smallest human DNA virus with 3200bp in a partially double-stranded circular DNA Lin et al. [1] and Lau et al. [2]. It has four partially overlapping open reading frames encoding polymerase/reverse transcriptase (RT), surface protein, core protein and X protein (HBx) respectively. Replication of HBV occurs through a reverse-transcribed RNA (Ribonucleic acid) intermediate by a reverse transcriptase enzyme that is without any proofreading capabilities leading to a highly error-prone nucleotide synthesis. This translates to high genetic variability that has led to the emergence of ten HBV genotypes (A-J) with forty sub-genotypes Ambachew et al. [3] Valsamakis [4] Feitelson, et al. and [5] Kramvis et al. [6]. According to Ambachew et al. [3] Kramvis et al. [7] and Ganem et al. [8] an estimated 250 million humans are chronically infected with HBV with an estimated 887,000 annual deaths occuring due to liver cirrhosis and hepatocellular carcinoma Guvernir et al. [9] In terms of mortality rates, HBV-related disease is ranked ninth and as fifth most important infectious agent Pourkarim et al. [10] and Szmaragd et al. [11].

Genotypes and subgenotypes of HBV show different geographical distributions with Genotype A being mainly found in Europe and Africa with subgenotype A2 in Europe and A1 in Africa respectively Norder et al. [12]. Genotypes B and C have been reported in Asia Norder et al. [12] and Ding et al. [13] while Genotype D has been found to be prevalent in Africa, Europe, the Mediterranean region, and India. Genotype E is found in both West and South Africa while Genotype F is predominant in South and Central America. Genotype G has been reported in France and the United States whereas Genotype H is present in Mexico and Central America Norder et al. [12]. Genotype I was isolated in Vietnam and Laos whereas the newest HBV genotype J was reported in the Ryukyu Islands of Japan, Huy et al. 2008 [14]. The prevalence rates of the various genotypes globally have been reported to be as follows; C (26%), D (22%), E (18%), A (17%), B (14%), F to I <2% by Velkov et al. [15]. Hepatitis B virus prevalence is highest in Sub-Saharan Africa with the viral genotypes having distinct geographic distributions and outcomes, Ambachew et al. [3] with genotypes A, D, and E found in diverse geographical locale. However, there is limited information on prevalent genotypes in many African countries Kramvis et al. [16].

The data from this study demonstrates the importance of genotyping as a guide on tailored treatment and the role of monitoring of biomarkers among CHB patients so as to improve disease out-come. Studies have shown that all genotypes respond to treatment with reverse transcriptase inhibitors, under interferon-α treatment with genotypes A and B showing an increased virological response and higher anti-HBe seroconversion amongst all Lin et al. [1] Velkov et al. [15] and Liu et al. [17]. Data on local genotype distribution is therefore critical in determining patient treatment and management options. In the present study, we therefore sought to investigate the prevalence rates of the various HBV genotypes and their biomarkers among hepatitis B patients chronically infected with HBV receiving treatment at a leading referral hospital in Western Kenya.

## 2 Materials and methods

### 2.1 Study design

A purposive approach was used to identify eligible records of patients receiving care at the Moi Teaching and Referral Hospital (MTRH) Gastro intestinal (GI) clinic. Census sampling was conducted to enroll study participants within the period starting from 1^st^ December 2019 to 31^st^ March 2020. Eligible patients were requested to provide a written informed consent to participate in the study.

### 2.2 Sample size calculation

The Cochran sample size determination formulae, Cochran [18], for populations >10,000 was used in the calculation of the sample size as follows;

$$n_{0}=\frac{Z^{2}*p*(1-p)}{e^{2}}$$

where;

Z = Standard normal deviation set at 1.96, corresponding to 95% confidence interval.

p = proportion of the population presumed to have the characteristic of study. A prevalence of 10% was used based on studies by Songok et al. [19] conducted in out-patients clinics in the North Rift, Kenya‥

q = 1 –p.

e = desired level of precision (i.e., the margin of error), set at 0.05

n_0_ = 138

The sample size was further reviewed for finite population (< 10,000) using the following modified Cochran’s formula.


$$n=\frac{n_{0}}{1+\frac{\left(n_{0}-1\right)}{N}}$$

Where;

- n_0_ = Cochran’s sample size recommendation
- N = The population size for finite populations (88)
- n = The new, modified sample size = 54 participants

The new sample size after modification was 54 participants.

### 2.3 Eligibility criteria

All adult CHB patients over 18 years of age with a history of HBsAg positivity of not less than six months met the initial inclusion criteria, however, among them, all women who were either pregnant and those who returned a negative Anti-HBc test result were excluded.

### 2.4 Study population and recruitment

Out of the initial 83 HBV positive patients, a total of 43 participants were found to be fully eligible and were enrolled.

### 2.5 Demographic data collection

Patient’s information including age and gender were abstracted from the patients’ files, transcribed into data collection forms and entered into MS office Excel sheets.

### 2.6 Sample collection and processing

Equal volumes (4mls) each of blood were collected separately into plain tubes and ethylenediaminetetraacetic acid (EDTA) tubes respectively from which serum was separated and aliquoted into a cryovial tubes for the analysis of serological markers whereas plasma was separated and aliquoted into two tubes for HBV genotyping and viral load determination respectively. Samples were stored at -20^օ^C until analysis.

### 2.7 Determination of virological markers

Serum samples from all patients were re-tested for HBsAg to confirm HBV positivity and screened for anti-HBc IgG for chronicity. Those with detectable anti-HBc and HBsAg levels were tested for HBeAg using a commercial enzyme-linked immunosorbent assay (ELISA) kit using Roche Fully Automatic Immunoassay Analyzer (Cobas e411, Random Assay, Luminescence, India).

### 2.8 DNA extraction

HBV viral DNA was extracted from 300μL of the patients’ plasma according to the manufacturer’s instructions using the Life River Viral DNA Isolation Kit (Shanghai ZJ Biotech, China). DNA was eluted in 50μL of elution buffer and stored at −20°C until use.

#### 2.8.1 Determination of HBV DNA viral load

The HBV DNA levels (viral load) were assayed using commercial kit HBV Real-TM Quant (Sacace Biotechnologies s.r.l., Como, Italy), for the quantitative detection of Hepatitis B Virus in human plasma following the manufacturers protocol. In brief, 10μl of extracted DNA was added to 50μl lyophilized master mix and loaded on the RT Rotor gene Q instrument (Qiagen Sciences LLC, German) for amplification following the manufacturer’s instructions.

### 2.9 HBV genotyping

The HBV DNA genotyping system based on real-time PCR amplification of HBV DNA using Sacace™ HBV Genotype A, B, C, D kit (Sacace Biotechnologies s.r.l., Como, Italy) was used for amplification of the pathogen genome specific region with specific HBV genotype primers. The amplified product was detected using fluorescent dyes. HBV DNA genotyping was then done using 10μL of DNA added to the 15μL master mix mixture of HBV-genotype-FRT for qualitative amplification of genotype A, B, C and D as recommended by the protocol using Rotor Gene Q equipment (Qiagen Sciences LLC, German).

### 2.10 Data analysis

STATA version 16 software was used to analyze quantitative data using the two variables to determine the relationship between the dependent (HBV genotype) and the independent variable (age, gender, HBV DNA viral load and HBeAg). Categorical variables were summarized using frequency tables, class intervals and percentages, whereas continuous data was summarized using means and standard deviations (SD). Values with p ≤ 0.05 were considered as statistically significant.

### 2.11 Ethical statement

This study was approved by MTRH/MOI University IREC (Ref: IREC/2019/246 and Approval No. 0003476) and the Moi Teaching and Referral Hospital facility management (Ref Number: ELD/MTRH/R&P/10/2/V.2/2010).

## 3 Results

### 3.1 Confirmation of chronic HBV patients

Out of the 83 HBV positive patients at the MTRH gastrointestinal clinic, 45 (54%) met the initial eligibility criteria by returning a positive test result for HBsAg (See Table 1).

**Table 1:** Confirmatory screening results of HBV based on HBsAg.

Variable	Category	Frequency	Percentage
**HBsAg**	Detectable	45	100
	Not detectable	0	0
	**Totals**	**45**	**100**

Of these, 43 (95.6%) of them returned a positive test result for anti-hepatitis B core Antibody (Anti-HBc) confirming chronic HBV infection (Table 2).

**Table 2:** Confirmatory screening results of HBV chronicity on anti-HBc (IgG).

Variable	Category	Frequency	Percentage
**Anti-HBc (Ig G)**	Detectable	43	95.6
	Not detectable	2	4.4
	**Totals**	**43**	**100**

### 3.2 Demographic characteristics of study participants

#### 3.2.1 Age

**Fig 1:**
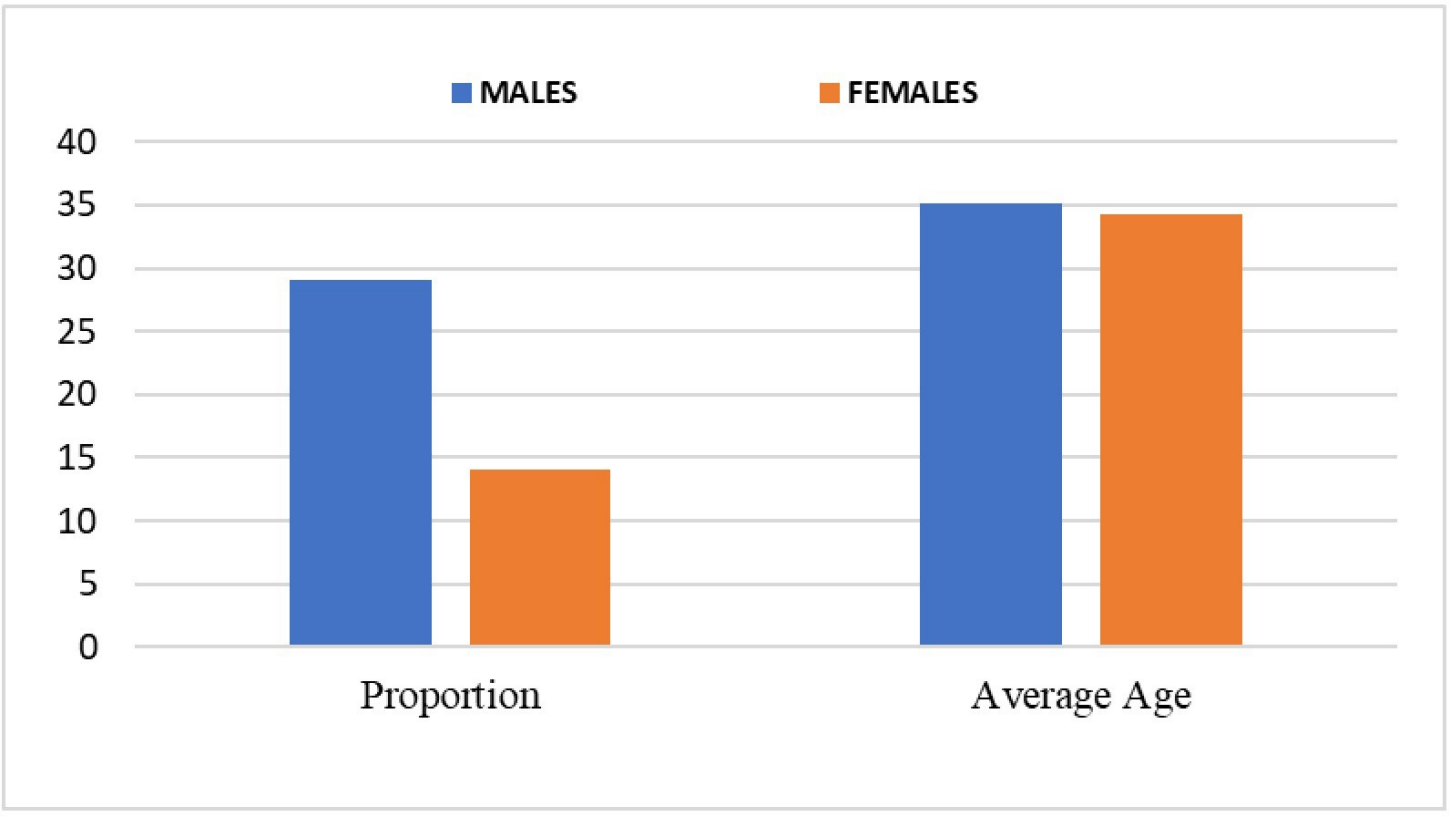
Showing demographic data of the gender and age in chronic HBV patients attending MTRH liver clinic.

The participants age ranges were between 21 to 61 years with a mean age of 34.8±10.2. With respect to gender, no difference was observed with means of 35.1±10.8 and 34.3±9.3 among male and female participants respectively (p = 0.7999) (see Fig 1).

#### 3.2.2 Gender

As shown in Fig 3 below, there were more males (n = 29,67.4%) than females (n = 14,32.6%) enrolled in the study translating to 2:1 ratio.

#### 3.2.3 Age frequency distribution (in years) amongst male and female participants

**Fig 2:**
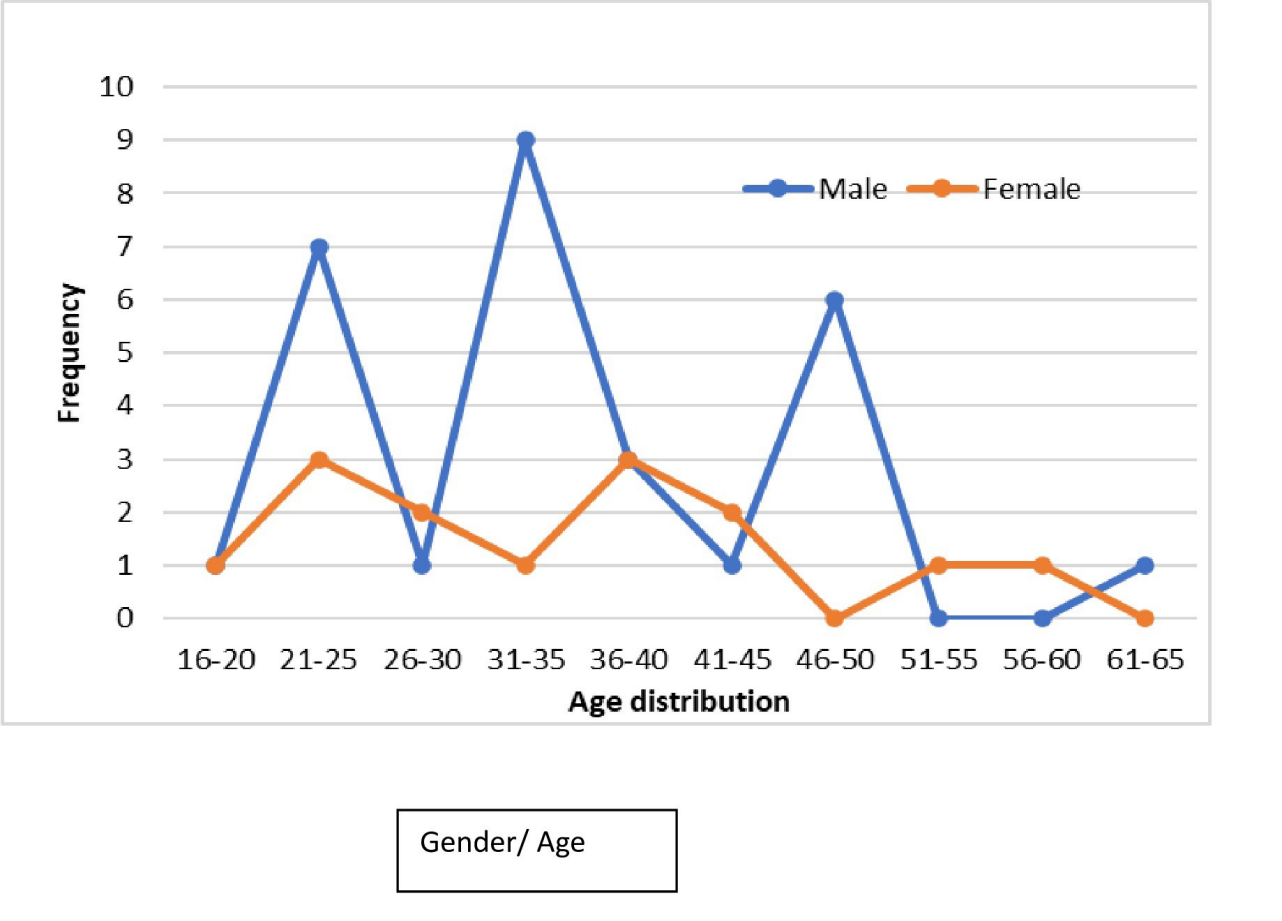
Age group frequency distribution (in years) amongst male and female participants.

As shown in Fig 2, variation in distribution across different age groups was observed more predominantly within the male gender where spikes were noted in age sets between 21–25, 31–35 and 46–50 with the highest spike occurring between the age group of 31–35 years. Additionally, the female gender showed a fairly well distributed curve amongst all the age groups.

### 3.3 Prevalence of HBV genotypes among the study participants

**Fig 3:**
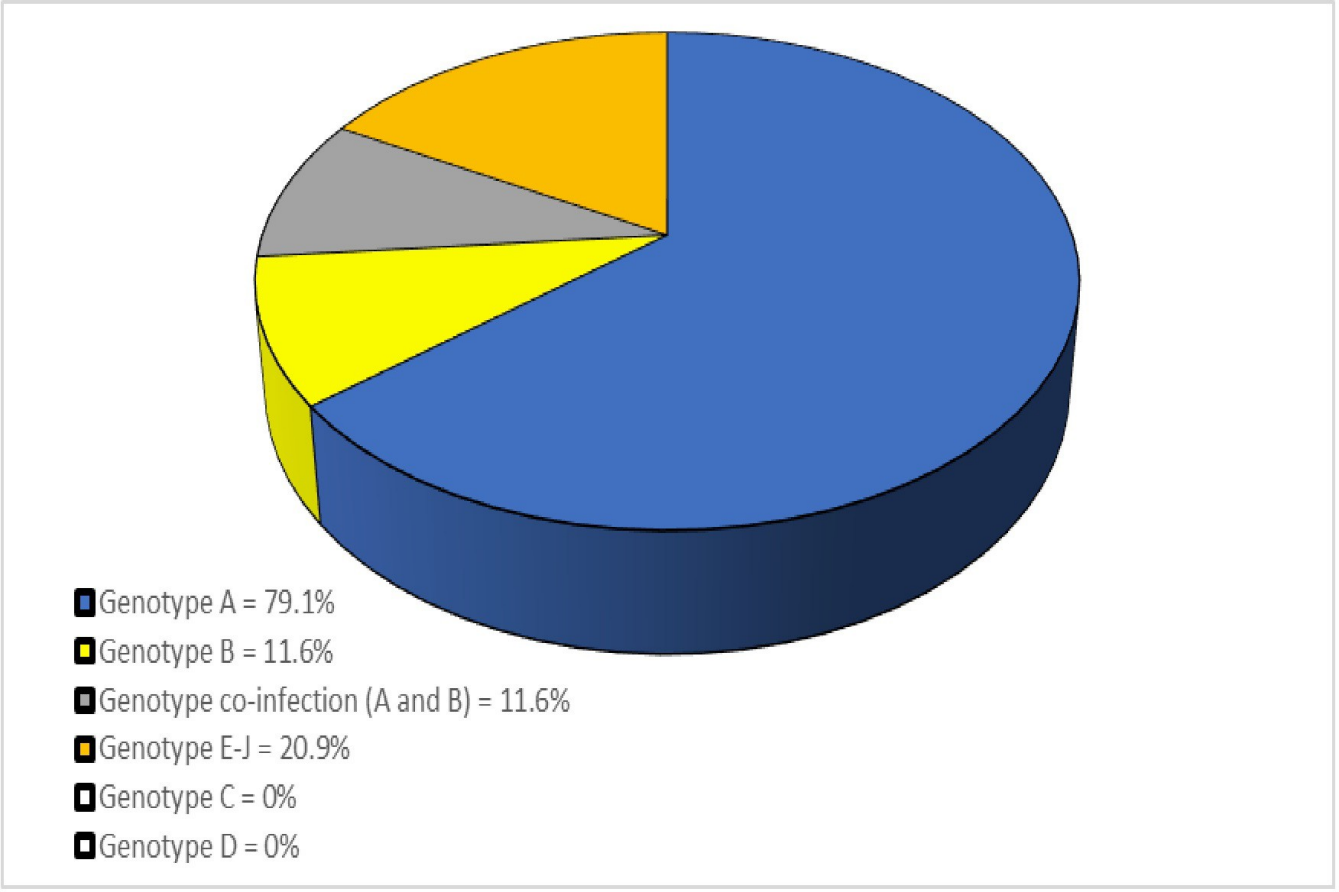
Showing chronic HBV genotypes and their prevalence among study participants. Note: The co-infection has been represented independently and as a single entity with the focus of narrating the circulating genotypes percentage and percentage of the co-infection thus the total percentage is above 100% (123.2%).

Most of the samples analyzed (79.1%) were found to have HBV genotype A, whereas another 11.6% had co-infection of HBV genotype A and B. The genotype C and D were undetected (0%) while 20.9% had the other un-characterized genotypes E-J (see Fig 3).

#### 3.3.1 Prevalence of various HBV genotypes among the male and female genders

**Fig 4:**
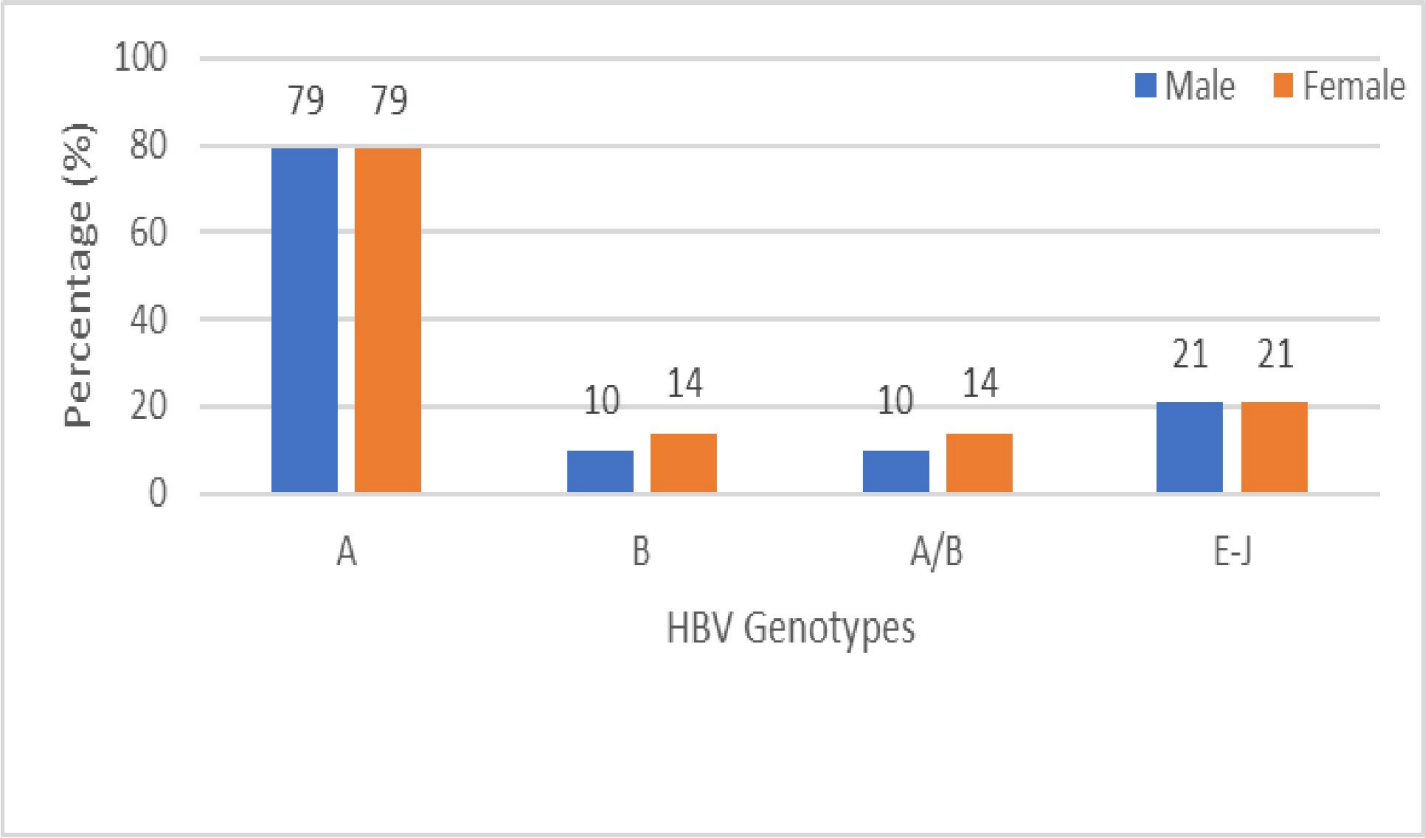
Chronic HBV genotypes and their prevalence among study participants.

The prevalence of HBV genotype A was the highest and evenly distributed (79%) in both genders. Co-infection with genotype A and B (genotype A/B) was more prevalent among females (14%) than males (10%) whereas the uncharacterized genotypes (E-J) were evenly distributed at 21% each in both the male and female (Fig 4).

#### 3.3.2 Distribution of HBV genotypes across various age groups

Spikes of high prevalence were noted among the age groups 31–35 and 46–50 years for male participants with genotype A, whereas the female gender had a fairly even distribution among all genotypes with a slightly higher distribution of genotype A/B between the age of 26–30 and 51–55 years.

### 3.4 Relationship between virological marker levels (HBV viral load and HBeAg) with the various HBV genotypes

#### 3.4.1 HBV viral load versus HBV genotypes

Table 3 below, shows the variation of HBV viral loads with HBV genotypes. Data shows that all genotypes presented with mostly detectable HBV DNA viral load (86%) with a higher proportion being genotype A (96.6%) followed by genotype A/B at 80% and finally genotype E-J at 55.6%. Genotype E-J also had a higher proportion on undetectable HBV DNA levels with a detection rate of 44.4%.

**Table 3:** Distribution of HBV viral load according to the genotypes among study participants.

		Genotype			
**Variable**	**Category**	**A** **(n = 29)**	**A/B** **(n = 5)**	**Other (E-J)** **(n = 9)**	**Total** **(n = 43)**
**HBV Viral Load**	Undetectable	1	1	4	**6**
	Detectable	16	1	4	**21**
	Extremely high	12	3	1	**16**

NB: The HBV viral load reference range (IU/mL) were categorized as following; Undetectable (<10), detectable (10–2000) and extremely high (>2000).

#### 3.4.2 HBeAg versus HBV genotypes

The distribution of HBeAg positivity was analyzed and compared across participants based on their HBV genotypes. The results are summarized in Table 4 below.

**Table 4:** Distribution of HBeAg according to the genotypes among study participants.

		Genotype			
**Variable**	**Category**	**A** **(n = 29)**	**A/B** **(n = 5)**	**Other (E-J)** **(n = 9)**	**Total** **(n = 43)**
**HBeAg**	Negative	23	4	8	**35**
	Positive	4	1	0	**5**
	Extremely High	2	0	1	**3**

Optical Density (OD) measurement was determined and used to categorize samples as being HBeAg negative (<1.0), positive (1–100) and extremely high (>100). As shown in the Table 4, a majority of participants (81.4%) presented with HBeAg negativity with the highest being the uncharacterized genotypes, E-J higher at 88.8% followed by genotype A/B at 80% and lowest being genotype A at 79.3%. Notably, 21% of participants with genotype A presented with slightly elevated HBeAg positivity whereas genotype A/B and E-J had 20% and 11.1% respectively.

## 4 Discussion

The present study sought to establish the prevalence and characteristics of the various HBV genotypes and their biomarkers among hepatitis B patients chronically infected with HBV attending Moi Teaching and Referral Hospital (MTRH) liver / gastrointestinal (GI) clinic between 1^st^ December 2019 to 31^st^ March 2020.

### 4.1 Demographic characteristics related with the various HBV genotypes among the study participants

#### 4.1.1 Age

The age ranged between 18–61 years, mean of 34.8 ± 10.2 with no significant difference between mean age in gender (p-Value = 0.7999). This findings compared well with a Kenyan study by Ochwoto et al. [20] which enrolled patients seeking medical services at hospitals throughout Kenya with the mean age was 36.5 ± 11.2 with ages ranging from 16–64 years. Our findings however contrasted those from another Kenyan study conducted in the coastal city of Mombasa by Kasera et al. [21] which included all HAV and HBV infected participants and therefore had a much younger population overall with age range between 4 months and 75 years old, mean of 23.6 ± 17.3, with; men 25.1 ± 19.2 years old and females 22.4 ± 15.5. Previous studies have reported the leading risk factors for HBV infection in our region (Western Kenya) to include sexual activity, tattoos and traditional marks and traditional circumcision [22]. It is also worth noting that the role of drug abuse, in driving infection rates which mostly affects younger people[23], is much lower in the western and other parts part of the country when compared to the coastal city of Mombasa.

With respect to the distribution of various genotypes in the population, our findings indicate that women had a fairly even genotype distribution across all ages whereas males presented with variations of spikes with genotype A peaking at age ranges 31–35 and 46–50 years. The reasons behind this variation in distribution remains to be determined. The study by Kasera et al. [21] found a high level of infection at the age groups 25–38 years old across gender (P < 0.022) followed by age group 39.0–52.0 years.

#### 4.1.2 Gender

Our findings appear to be in support of other Kenyan studies that have shown a preponderance of HBV infection rates among males when compared to females, Mabeya et al. [23] and Kasera et al. [21]. Based on our data, the male to female ratio was at 2:1 and was very similar with a Kenyan study by Ochwoto et al. [20] with a 2:1 ratio. Interestingly, there was no gender predominance across all genotypes except a slightly higher representation of co-infection with genotype A/B among the females’ participants at 58%.

#### 4.1.3 Prevalence rates of the various HBV genotypes among the study participants

As expected, genotype A was the most predominant at 79.1%. However, when compared to other studies conducted in Kenya, the prevalence rate is much lower. Studies by Songok et al. [19] and Koech et al [24] found genotype A to have prevalence rate of 93% which was very close that that obtained by Ochwoto et al. [20] where genotype A had a prevalence rate of 90.3%. This may be suggestive of increase in the prevalence rates of other genotypes in Kenya. Indeed, based on our study findings, an unexpected co-infection rate of 11.6% was observed for genotype A/B raising concerns of a possible new phenomenon since genotype B is associated with vertical (mother to child) transmission as stated by Kafeero et al. [25] and Guo et al. [26]. Further, a relatively high proportion (20.9%) was noted to have other genotypes not uncharacterized in the present i.e. E-J which is uncharacteristic of African populace.

Other studies also demonstrate that the actual concentration of HBV DNA plays a crucial role in identification of the genotypes and the patients should be followed up for genotyping after two months Baclig et al. [27].

### 4.2 Characterization of HBV genotypes based on virological markers among study participants

Serological markers for HBV infection studied include HBsAg, anti-HBs, HBeAg, anti-HBe, and anti-HBc IgM and IgG. The identification of serological markers allows: to identify patients with HBV infection; elucidate the natural course of chronic hepatitis B; assess the clinical phases of infection; and eventually monitor antiviral therapy Song et al. [28] and Control, et al. [29].

#### 4.2.1 HBV Viral load marker

All genotypes presented with mostly detectable HBV DNA viral load (86%) with a higher proportion being genotype A (96.6%). However, it was also noted that among participants with co-infection with genotypes of A/B, a large proportion presented with HBV DNA levels of >20,000IU/ml which is indicative that the virus is active and has the greatest potential to cause damage to the patients’ liver. Among the uncharacterized genotype (E-J) a lower percentage of participants had HBV DNA levels >20,000IU/ml. A comparison with viral load on alarming levels (>20,000IU/ml) requiring medical attention is in agreement with a study by Elizalde et al. [30] and Seeger [31] where high viral load levels and the HBeAg-positive have been identified as markers associated with the risk of liver disease progression as stated. Further, a slightly lower percentage of the undetectable HBV DNA was noted in the current study and is in agreement with a study by Baclig et al. [27] which suggested that the portion showing 24% of CHB patients had non-detectable levels be recommended for follow-up testing for HBV DNA detection and quantitation within two months to reduce the increased risk of liver cirrhosis.

This is in comparison with a strong discussion made in a study by Wong et al. [32] suggesting that Intrahepatic HBV DNA was found to be of clinical significance in several aspects including significant correlation with the degree of fibrosis and also as observed in at least one patient, intrahepatic HBV DNA was still present even when serum HBV DNA was no longer detectable. This supports the previous findings that for Asian HBV carriers, there is no cut off HBV DNA value below which disease progression would not occur. There is a strong correlation between serum and intrahepatic HBV DNA levels, suggesting that serum HBV DNA levels can be used as an indication of intrahepatic HBV DNA levels.

#### 4.2.2 HBeAg marker

A majority of participants in the present study presented with HBeAg negativity at 81.4% with genotypes E-J being the highest at 88.8%. However, as compared to previous findings, there is a slightly higher representation of HBeAg positivity across all genotypes that could be of importance as since high viral load levels and the HBeAg-positive status have been identified as markers associated with the risk of liver disease progression Elizalde et al. [30]. This finding strongly agrees with a study by Chandra et al. [33] and Lok et al. [34] which demonstrated the presence of viral replication leading to the high viral load among patients even in the absence of HBeAg secretion due to the emergence of HBeAg negative G1862 mutation and continue to have ongoing liver disease. As recommended by Bárcena Marugán et al. [35], HBeAg status should be checked every 6–12 months with patients who remain HBeAg-positive with HBV DNA > 20 000 IU/mL after 3–6 months and are aged < 40 years old, being considered for liver biopsy.

## 5 Conclusions

Among female participants, a fairly even genotype distribution was observed across all ages in the study population. There was a high variability in genotype representation across various age groups among the male gender especially with genotype A. The highest peak was observed in the age group of 31–35 years followed by 46–50 years. There was no gender predominance across all genotypes except with genotype A/B where females predominated at 58%. However, the most predominant genotype observed across each gender was genotype A. Genotypes C and D were undetected. HBV DNA was detectable in a majority of samples from all the genotypes with a higher detection rate observed in genotype A. Additionally, a majority of participants presented with HBeAg negativity with participants in genotype E-J being highest.

## Supporting information

S1 File (DOC)
