# Supplementary material for: Prevalence of HBV genotypes among patients attending Moi Teaching and Referral Hospital liver clinic
Source: PLoS One. 2024 Jul 10;19(7):e0305753. doi: 10.1371/journal.pone.0305753 (PMC11236169; doi:10.1371/journal.pone.0305753)
Supplement: S1 File — (DOC) [file pone.0305753.s001.doc]

| **Prevalence of HBV Genotypes among Patients Attending Moi Teaching and Referral Hospital Liver Clinic** | | | | | | | | |
| --- | --- | --- | --- | --- | --- | --- | --- | --- |
| ***NUMBER*** |  | ***AGE*** | ***SEX*** | ***HBsAG*** | ***Anti-HBc*** | ***HBeAG*** | ***HBV Viral Load*** | ***GENOTYPE*** |
| 1 |  | 1993 | F | Positive | Positive | 0.113 | 882.21 IU/mL | A |
| 2 |  | 1985 | F | Positive | Positive | 0.222 | 442.30 IU/mL | A |
| 3 |  | 1987 | M | Positive | Positive | 0.708 | 709 IU/mL | A |
| 4 |  | 1977 | M | Positive | Positive | 0.144 | 41.53 IU/mL | A |
| 5 |  | 2000 | M | Positive | Positive | 0.126 | 1,745 IU/mL | A |
| 6 |  | 1990 | M | Positive | Positive | 0.106 | 509 IU/mL | A |
| 7 |  | 1976 | M | Positive | Positive | 0.13 | 800,922 IU/mL | A |
| 8 |  | 1985 | M | Positive | Positive | 0.436 | 5,999.08 IU/mL | A |
| 9 |  | 1984 | M | Positive | Positive | 0.469 | 153,015.05 IU/mL | A |
| 10 |  | 1975 | M | Positive | Positive | 0.748 | 353,366.38 IU/mL | A |
| 11 |  | 2004 | F | Positive | Positive | 0.201 | 177.21 IU/ml | A |
| 12 |  | 1998 | F | Positive | Positive | 4.65 | 2,320 IU/mL | A |
| 13 |  | 2000 | M | Positive | Positive | 388.5 | 4,711.09 IU/mL | A |
| 14 |  | 1988 | M | Positive | Positive | 0.262 | 3,967.26 IU/mL | A |
| 15 |  | 1977 | M | Positive | Positive | 0.801 | 700.40 IU/mL | A |
| 16 |  | 1982 | F | Positive | Positive | 0.423 | 37.45 IU/mL | A |
| 17 |  | 1999 | M | Positive | Positive | 0.502 | 695.66 IU/mL | A |
| 18 |  | 2003 | M | Positive | Positive | 0.145 | 90,041.62 IU/mL | A |
| 19 |  | 1989 | F | Positive | Positive | 368.4 | 841.27 IU/mL | A |
| 20 |  | 1966 | F | Positive | Positive | 28.77 | 4347.95 IU/mL | A |
| 21 |  | 1992 | M | Positive | Positive | 0.121 | 157,322 IU/mL | A |
| 22 |  | 2002 | M | Positive | Positive | 0.235 | 122.33 IU/mL | A |
| 23 |  | 1989 | M | Positive | Positive | 0.677 | 5,021 IU/mL | A |
| 24 |  | 1999 | F | Positive | Positive | 0.236 | 146.77 IU/mL | A |
| 25 |  | 1988 | M | Positive | Positive | 0.769 | < 10 IU/mL | A |
| 26 |  | 1989 | M | Positive | Positive | 0.109 | 301.77 IU/mL | A |
| 27 |  | 1961 | M | Positive | Positive | 29.18 | 7,200.09 IU/mL | A |
| 28 |  | 1982 | F | Positive | Positive | 0.686 | 177.20 IU/mL | A |
| 29 |  | 1990 | M | Positive | Positive | 52.28 | 1,447.72 IU/ml | A |
| 30 |  | 1972 | F | Positive | Positive | 0.4 | 2,785 IU/mL | A/B |
| 31 |  | 1980 | M | Positive | Positive | 0.134 | 1,200.09 IU/mL | A/B |
| 32 |  | 1999 | M | Positive | Positive | 27.66 | 4,210.01 IU/mL | A/B |
| 33 |  | 1998 | M | Positive | Positive | 0.701 | 2,777.09 IU/mL | A/B |
| 34 |  | 1995 | F | Positive | Positive | 0.298 | < 10 IU/mL | A/B |
| 35 |  | 1998 | M | Positive | Positive | 1232 | < 10 IU/mL | E-J |
| 36 |  | 1977 | M | Positive | Positive | 0.778 | 991.79 IU/mL | E-J |
| 37 |  | 1987 | F | Positive | Positive | 0.402 | 6,221.03 IU/mL | E-J |
| 38 |  | 1987 | F | Positive | Positive | 0.246 | < 10 IU/mL | E-J |
| 39 |  | 1989 | M | Positive | Positive | 0.169 | 1,345.34 IU/mL | E-J |
| 40 |  | 1995 | M | Positive | Positive | 0.463 | < 10 IU/mL | E-J |
| 41 |  | 2000 | F | Positive | Positive | 0.507 | 727.76 IU/mL | E-J |
| 42 |  | 1988 | M | Positive | Positive | 0.35 | < 10 IU/mL | E-J |
| 43 |  | 1976 | M | Positive | Positive | 0.615 | 399.87 IU/mL | E-J |
|  |  |  |  |  |  |  |  |  |
